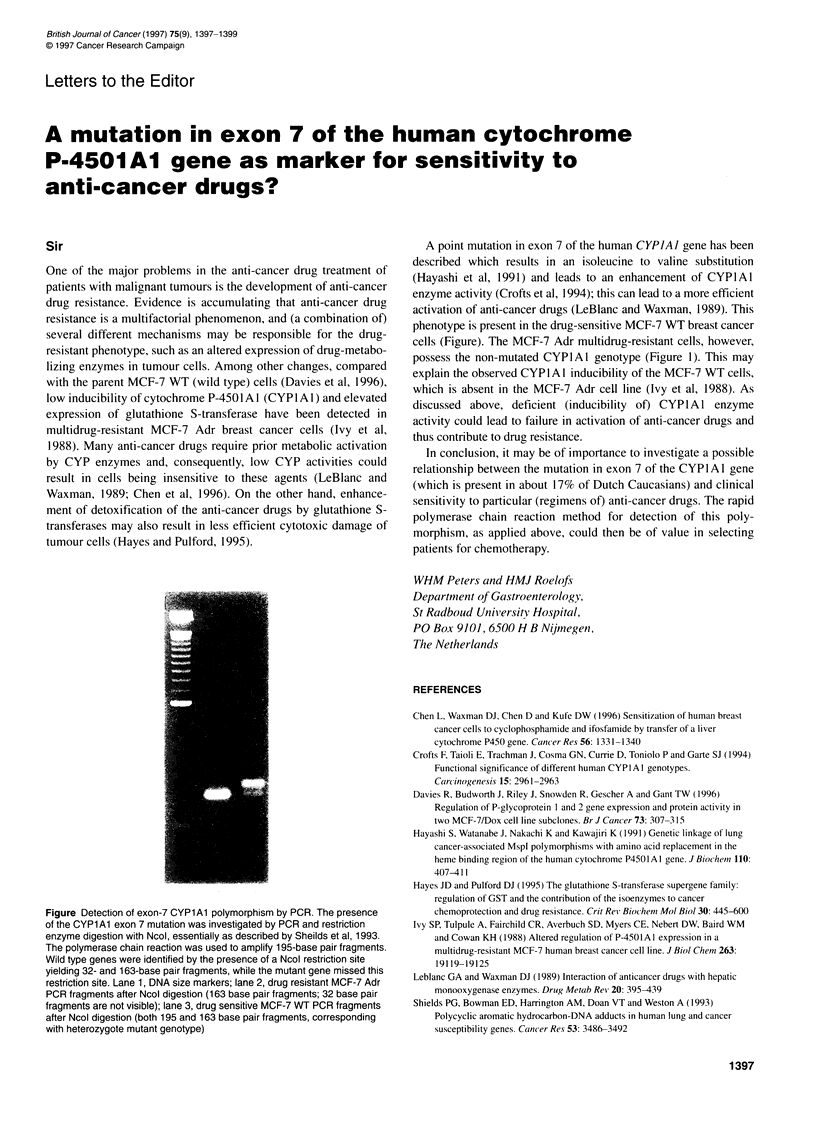# A mutation in exon 7 of the human cytochrome P-4501A1 gene as marker for sensitivity to anti-cancer drugs?

**DOI:** 10.1038/bjc.1997.237

**Published:** 1997

**Authors:** W. H. Peters, H. M. Roelofs

## Abstract

**Images:**


British Journal of Cancer (1997) 75(9), 1397–1399
© 1997 Cancer Research Campaign

Letters to the Editor

### A mutation in exon 7 of the human cytochrome P-4501A1 gene as marker for sensitivity to anti-cancer drugs?

**Sir**

One of the major problems in the anti-cancer drug treatment of patients with malignant tumours is the development of anti-cancer drug resistance. Evidence is accumulating that anti-cancer drug resistance is a multifactorial phenomenon, and (a combination of) several different mechanisms may be responsible for the drug-resistant phenotype, such as an altered expression of drug-metabolizing enzymes in tumour cells. Among other changes, compared with the parent MCF-7 WT (wild type) cells (Davies et al, 1996), low inducibility of cytochrome P-4501A1 (CYP1A1) and elevated expression of glutathione S-transferase have been detected in multidrug-resistant MCF-7 Adr breast cancer cells (Ivy et al, 1988). Many anti-cancer drugs require prior metabolic activation by CYP enzymes and, consequently, low CYP activities could result in cells being insensitive to these agents (LeBlanc and Waxman, 1989; Chen et al, 1996). On the other hand, enhancement of detoxification of the anti-cancer drugs by glutathione S-transferases may also result in less efficient cytotoxic damage of tumour cells (Hayes and Pulford, 1995).

**Figure** Detection of exon-7 CYP1A1 polymorphism by PCR. The presence of the CYP1A1 exon 7 mutation was investigated by PCR and restriction enzyme digestion with NcoI, essentially as described by Sheilds et al, 1993. The polymerase chain reaction was used to amplify 195-base pair fragments. Wild type genes were identified by the presence of a NcoI restriction site yielding 32- and 163-base pair fragments, while the mutant gene missed this restriction site. Lane 1, DNA size markers; lane 2, drug resistant MCF-7 Adr PCR fragments after NcoI digestion (163 base pair fragments; 32 base pair fragments are not visible); lane 3, drug sensitive MCF-7 WT PCR fragments after NcoI digestion (both 195 and 163 base pair fragments, corresponding with heterozygote mutant genotype)

A point mutation in exon 7 of the human *CYP1A1* gene has been described which results in an isoleucine to valine substitution (Hayashi et al, 1991) and leads to an enhancement of CYP1A1 enzyme activity (Crofts et al, 1994); this can lead to a more efficient activation of anti-cancer drugs (LeBlanc and Waxman, 1989). This phenotype is present in the drug-sensitive MCF-7 WT breast cancer cells (Figure). The MCF-7 Adr multidrug-resistant cells, however, possess the non-mutated CYP1A1 genotype (Figure 1). This may explain the observed CYP1A1 inducibility of the MCF-7 WT cells, which is absent in the MCF-7 Adr cell line (Ivy et al, 1988). As discussed above, deficient (inducibility of) CYP1A1 enzyme activity could lead to failure in activation of anti-cancer drugs and thus contribute to drug resistance.

In conclusion, it may be of importance to investigate a possible relationship between the mutation in exon 7 of the CYP1A1 gene (which is present in about 17% of Dutch Caucasians) and clinical sensitivity to particular (regimens of) anti-cancer drugs. The rapid polymerase chain reaction method for detection of this polymorphism, as applied above, could then be of value in selecting patients for chemotherapy.

*WHM Peters and HMJ Roelofs*
*Department of Gastroenterology,*
*St Radboud University Hospital,*
*PO Box 9101, 6500 H B Nijmegen,*
*The Netherlands*

**REFERENCES**

Chen L, Waxman DJ, Chen D and Kufe DW (1996) Sensitization of human breast cancer cells to cyclophosphamide and ifosfamide by transfer of a liver cytochrome P450 gene. *Cancer Res* **56**: 1331–1340

Crofts F, Taioli E, Trachman J, Cosma GN, Currie D, Toniolo P and Garte SJ (1994) Functional significance of different human CYP1A1 genotypes. *Carcinogenesis* **15**: 2961–2963

Davies R, Budworth J, Riley J, Snowden R, Gescher A and Gant TW (1996) Regulation of P-glycoprotein 1 and 2 gene expression and protein activity in two MCF-7/Dox cell line subclones. *Br J Cancer* **73**: 307–315

Hayashi S, Watanabe J, Nakachi K and Kawajiri K (1991) Genetic linkage of lung cancer-associated MspI polymorphisms with amino acid replacement in the heme binding region of the human cytochrome P4501A1 gene. *J Biochem* **110**: 407–411

Hayes JD and Pulford DJ (1995) The glutathione S-transferase supergene family: regulation of GST and the contribution of the isoenzymes to cancer chemoprotection and drug resistance. *Crit Rev Biochem Mol Biol* **30**: 445–600

Ivy SP, Tulpule A, Fairchild CR, Averbuch SD, Myers CE, Nebert DW, Baird WM and Cowan KH (1988) Altered regulation of P-4501A1 expression in a multidrug-resistant MCF-7 human breast cancer cell line. *J Biol Chem* **263**: 19119–19125

Leblanc GA and Waxman DJ (1989) Interaction of anticancer drugs with hepatic monooxygenase enzymes. *Drug Metab Rev* **20**: 395–439

Shields PG, Bowman ED, Harrington AM, Doan VT and Weston A (1993) Polycyclic aromatic hydrocarbon-DNA adducts in human lung and cancer susceptibility genes. *Cancer Res* **53**: 3486–3492